# Patterns of brown bear damages on apiaries and management recommendations in the Cantabrian Mountains, Spain

**DOI:** 10.1371/journal.pone.0206733

**Published:** 2018-11-28

**Authors:** Javier Naves, Andrés Ordiz, Alberto Fernández-Gil, Vincenzo Penteriani, María del Mar Delgado, José Vicente López-Bao, Eloy Revilla, Miguel Delibes

**Affiliations:** 1 Department of Conservation Biology, Estación Biológica de Doñana, Seville, Spain; 2 Faculty of Environmental Sciences and Natural Resource Management, Norwegian University of Life Sciences, Ås, Norway; 3 Research Unit of Biodiversity (UMIB, UO-CSIC-PA), Oviedo University—Campus Mieres, Mieres, Spain; 4 Pyrenean Institute of Ecology (IPE), CSIC, Zaragoza, Spain; Liverpool John Moores University, UNITED KINGDOM

## Abstract

Large carnivores are often persecuted due to conflict with human activities, making their conservation in human-modified landscapes very challenging. Conflict-related scenarios are increasing worldwide, due to the expansion of human activities or to the recovery of carnivore populations. In general, brown bears *Ursus arctos* avoid humans and their settlements, but they may use some areas close to people or human infrastructures. Bear damages in human-modified landscapes may be related to the availability of food resources of human origin, such as beehives. However, the association of damage events with factors that may predispose bears to cause damages has rarely been investigated. We investigated bear damages to apiaries in the Cantabrian Mountains (Spain), an area with relatively high density of bears. We included spatial, temporal and environmental factors and damage prevention measures in our analyses, as factors that may influence the occurrence and intensity of damages. In 2006–2008, we located 61 apiaries, which included 435 beehives damaged in the study area (346 km^2^). The probability of an apiary being attacked was positively related to both the intensity of the damage suffered the year before and the distance to the nearest damaged apiary, and negatively related to the number of prevention measures employed as well as the intensity of the damage suffered by the nearest damage apiary. The intensity of damage to apiaries was positively related to the size of the apiary and to vegetation cover in the surroundings, and negatively related to the number of human settlements. Minimizing the occurrence of bear damages to apiaries seems feasible by applying and maintaining proper prevention measures, especially before an attack occurs and selecting appropriate locations for beehives (e.g. away from forest areas). This applies to areas currently occupied by bears, and to neighbouring areas where dispersing individuals may expand their range.

## Introduction

The trade-off between species conservation and the management of human-wildlife conflicts is central to conservation biology [1, 2]. Damages and risks caused by wildlife can be of emotional and economic importance, thus generating conflict and persecution by people [3]. This is especially true for large carnivores, whose conservation in human-dominated landscapes is increasingly challenging [4, 5, 6].

The occurrence of large carnivores near human settlements and infrastructures is frequently perceived as problematic because these animals can attack livestock and pets as well as damage crops and, although attacks on humans are very rare, especially in Europe [7], people can fear them [8]. Conflict-related scenarios are increasing particularly where large carnivore populations are re-occupying areas and habitats lost during the last few centuries, as well as where human activities are expanding [9, 10, 11]. In such scenarios, collecting baseline information on human-wildlife interactions is an essential step to delineate effective conflict mitigation strategies [9, 12, 13, 14].

Interactions between large carnivores and human activities are receiving increasing attention, and brown bears *Ursus arctos* are no exception. Bears generally avoid humans and their settlements, but some individuals use areas closer to people or anthropogenic infrastructures even if this behaviour is not always linked to food availability [15]. Nevertheless, the occurrence of bear damages in human-modified landscapes implies that anthropogenic food resources, such as beehives or livestock, are available [9, 16]. Yet, the association of damages with factors that predispose bears to such predation events have been rarely investigated (e.g. [17, 18, 19, 20]).

Beehives have long been present throughout most of the brown bear range in Eurasia and North America, and patterns of bear damages are heterogeneous [12, 19, 21, 22, 23, 24]. Therefore, understanding how different factors can influence predation patterns should help design appropriate measures to prevent and mitigate bear damages.

**Fig 1:**
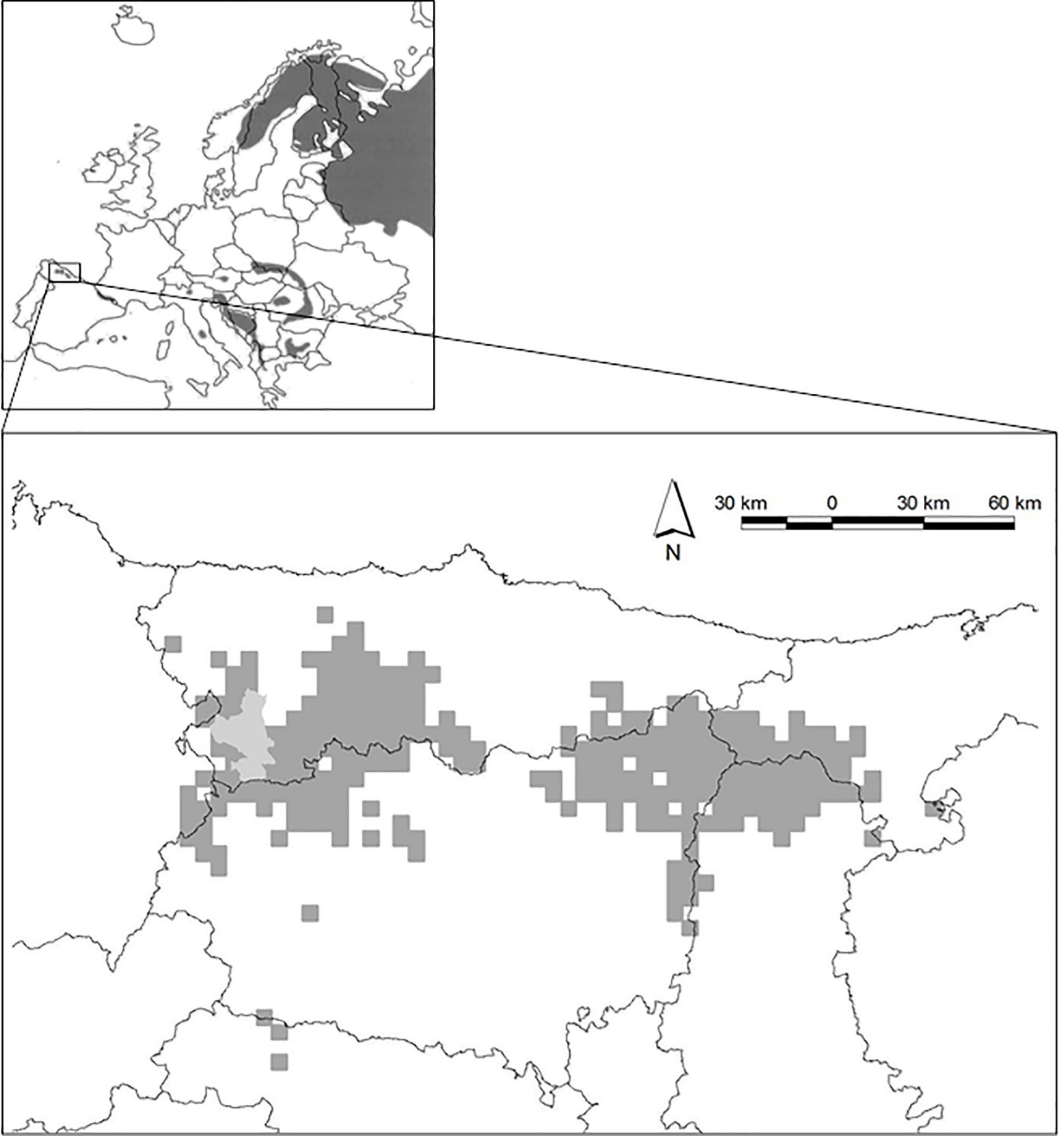
Location of the study area (346 km^2^; in light grey) within the current distribution of the brown bear in the Cantabrian Mountains (dark grey; according to Naves et al. 2003 [30]). Coordinates of centroid to the study area: 43^o^ 0.370’ N, 6^o^ 38.534’ W.

Due to habitat encroachment or reduction of bear habitats and chronic bear damages to human property, the isolated and critically endangered population of brown bears in the Cantabrian Mountains (Spain) provides the opportunity to investigate the factors related to bear-human conflict in this study area and elsewhere in Europe (Fig 1; [25, 26, 27]). The conflict scenario related to damages and the conservation of this bear population has been the subject of recent analyses [28], and this brown bear population has showed the highest rate of apiary (i.e., an aggregation of beehives spread over a given area) damages per bear in Europe [12]. Therefore, mitigating bear damages seems essential to promote bear-human coexistence and bear recovery in this area.

**Fig 2:**
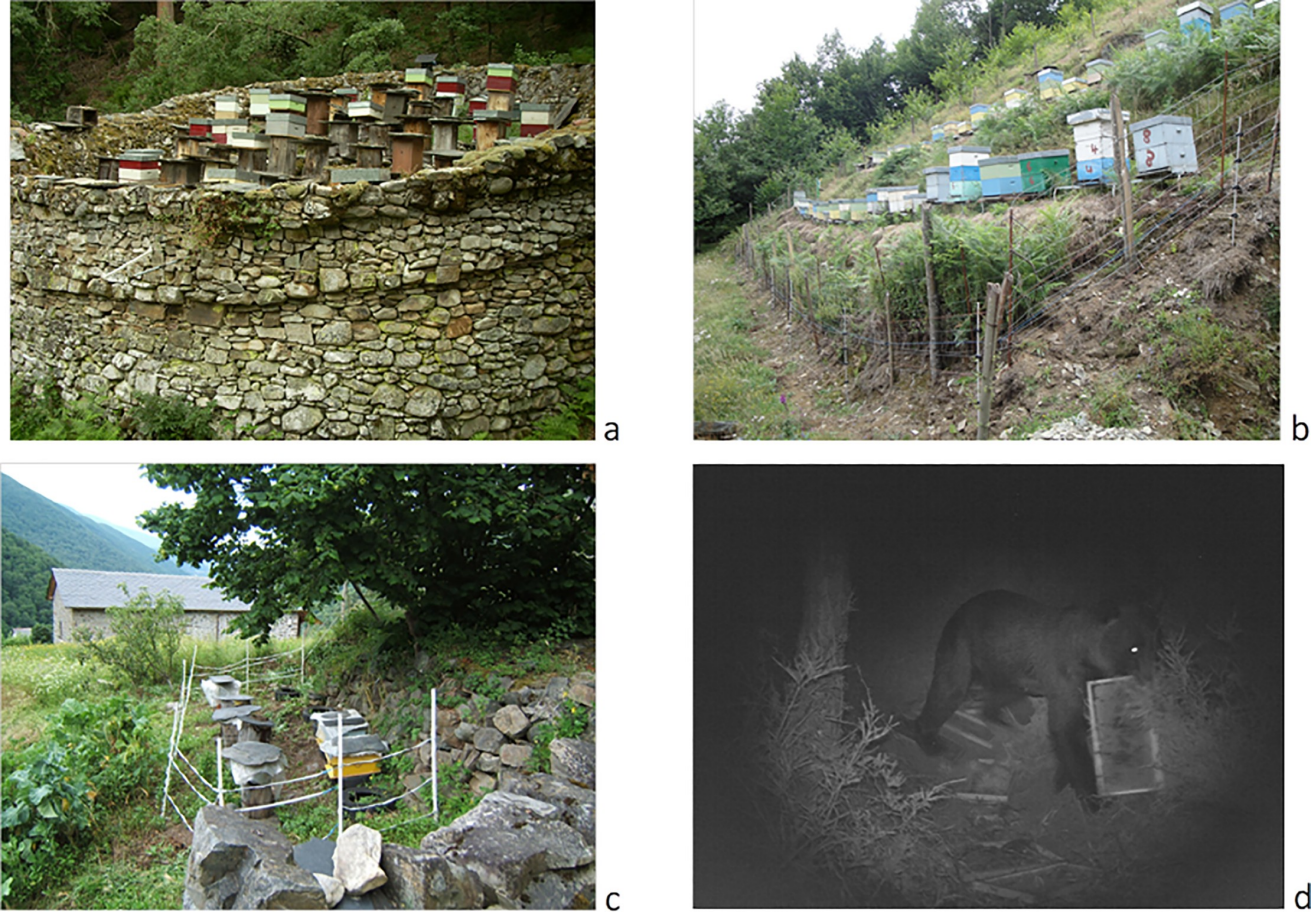
In the Cantabrian Mountains, apiaries have traditionally been protected by stonewalls called ‘cortines’ or ‘albarizas’ (a; picture: J. Naves). In recent times, the use of this ancient and traditional prevention method to deter bears has been lost in many areas; although some apiaries still have rock walls, several of them only use electric fences (b; picture: J. Naves) or combine both approaches (c; picture: A. Ordiz). Picture d (picture: A. Ramos, Principado de Asturias) shows the incursion of a brown bear in an apiary in the study area.

Our aim was to identify spatial and temporal correlates of bear damages to apiaries. In addition, we tried to identify environmental factors and prevention measures that may explain the occurrence and intensity of damages. We expected that the probability that a given apiary is damaged may be higher near to a damaged apiary and if the damage intensity to the latter is high. We therefore assumed that once a bear has obtained a positive reward (food), it may be prone to search for other apiaries nearby. We also expected that damage intensity to apiaries would vary over the years because of yearly co-occurrence of other factors such as the availability of food resources, spatial distribution of bears, or their space use and differential habitat selection. Furthermore, we expected that the probability that an apiary is damaged may depend on whether it has been damaged the year before and the intensity of the damage during the same year. We also considered a set of variables related to the characteristics (e.g., number of beehives, presence of damage prevention measures, including electric and/or traditional stonewalls, see Fig 2) and other local environmental correlates (such as vegetation cover, roads and human settlements) of the apiaries. We expected that apiaries with implemented preventive measures would have a lower probability of been damaged than unprotected ones, whereas denser vegetation cover in the proximity of apiaries would increase the probability of damages, because approaching the apiary would be less risky for bears. Finally, we expected that human infrastructures around apiaries would deter bears from approaching and eventually using apiaries, because risk exposure for bears may be directly related to human activities [15, 29].

## Methods

### The study area and the Cantabrian brown bear population

The Cantabrian Mountains are parallel to the Atlantic coast (Cantabrian Sea) of NW Spain. Mean tree species are beech *Fagus sylvatica*, oak *Quercus* spp., birch *Betula alba* and chestnut *Castanea sativa*. Beech and birch dominate in north face slopes and higher altitudes, over 600–700 m, whereas oak and chestnut are more abundant in south faces slopes and lower altitudes. Forests are interspersed with pasturelands and shrubs of broom *Cytisus* spp., *Genista* spp., heather *Erica* spp., *Calluna vulgaris*, and bilberry *Vaccinium myrtillus*, the latter usually dominating montane and subalpine levels (ca. 1,000–1,700 m a.s.l.; more details in [30, 31]).

Brown bears are distributed in two connected subpopulations [32], occupying ca. 7000 km^2^, with above 200 individuals in the western subpopulation (CI95% = 168–260 individuals) and 20 in the eastern subpopulation (CI95% = 12–40 individuals; [26]). In particular, we focused our analyses on a 346 km^2^ area located in the western Cantabrian Mountains (southwestern Asturias), including the municipalities of Cangas de Narcea, Ibias, and Degaña, with an elevation range between 450 and 1850 m a.s.l. (Fig 1). This study area includes portions of the Cantabrian Mountains with the highest bear densities, but also some peripheral areas (Fig 1). Among other economic activities such as livestock herding and mining, beekeeping in fixed apiaries is an important traditional activity in this area.

### Apiaries in the study area and environmental attributes

We used the official database of bear damage claims from the Regional Government of Asturias. The database is annual and for each claim, it includes: personal data of the claimer and the ranger that visited the place, county, record number, parish, village, Julian date, administrative situation and geographical coordinates, amount of money compensated, damaged species, and number of beehives attacked. We selected all the claims of bear damages that occurred in the study area between 2006 and 2008, and we visited all of the damaged beehives in the field in June and July 2009 to characterize them, e.g., to determine if they had protective measures and their type, and to collect environmental information on the surroundings of the apiaries. Additionally, we actively searched for additional apiaries in the study area, through direct observations and surveys. These apiaries were not included in the official dataset of bear damages, thus we considered them as the reference dataset of non-damaged apiaries.

In each of the visited apiaries, we collected information on several parameters (Table 1). At the apiary level, we recorded the number of beehives and the presence of damage prevention methods: electric fences, wire mesh fence and/or with stone walls, known as ‘cortines’ or ‘albarizas’, which are traditional constructions used to prevent the access of bears to beehives (Fig 2). We also compiled information on different environmental attributes that may influence the vulnerability of apiaries to bear attacks. Environmental attributes were recorded at three different spatial scales, within a 30, 500 and 2000 m radius around the apiaries. At the 30 m scale, we visually estimated vegetation cover as both the percentage of scrubland (bushy vegetation mainly composed by heathers and brooms, over 1 m in height), percentage of forest, and percentage cover of human infrastructures (buildings and paved and unpaved roads). Finally, we extracted the vegetation cover, number of settlements, and length of paved and unpaved roads within 500 and 2000 m radius plots around the central beehive of each apiary, using GIS layers from Cartografía Temática Ambiental of the Principado de Asturias (Hojas del Mapa de Vegetación, Litología, Roquedos y Hábitat del Oso. Escala 1:25000. Principado de Asturias, Spain).

**Table 1:** Variables recorded in the study of brown bear damages to apiaries in the Cantabrian Mountains, Spain.

Variables	Description	Values / Units
**1. Response variables**		
Probability of bear damage	Apiary damaged / not damaged by bears in a given year	binomial (0,1)
Intensity of bear damage	Number of damaged beehives (> 0) in a damaged apiary in a given year	count (1,2, …N)
**2. Predictors**		
*Effects of spatial closeness/damage intensity of neighbour apiaries on damaged apiaries*		
Distance nearest	Distance to the nearest damaged apiary in a given year	km
Intensity nearest	Number of damaged beehives (> 0) in the nearest damaged apiary in a given year	count (1,2, …N)
*Yearly patterns in damaged apiaries*		
Probability-1	Apiary damaged / not damaged the year before	binomial (0,1)
Intensity-1	Number of damaged beehives the year before	count (0,1,2, …N)
Year	Specific year of the study period (2006, 2007 or 2008)	categorical
*Apiary features*		
Prevention	Presence / absence of prevention measures (electric fences, wire mesh fence, stone walls)	binomial (0,1)
N_prevention	Combined number of apiary prevention measures	count (0,1,2,3)
N_beehives	Number of beehives in the apiary	count (1,2, …N)
*Landscape attributes surrounding the apiary*		
Forest_30	Percentage of forest cover within a 30 m radius area around the apiary	%
Scrub_30	Percentage of scrub (vegetation >1 m in height) cover within a 30 m radius area around the apiary	%
Human_30	Percentage of human settlements within a 30 m radius area around the apiary	%
Forest_500	Forest cover within a 500 m radius area around the apiary	ha
Forest_2000	Forest cover within a 2000 m radius area around the apiary	ha
Infrastructures_500	Length of paved and unpaved roads within a 500 m radius area around the apiary	km
Infrastructures_2000	Length of paved and unpaved roads within a 2000 m radius area around the apiary	km
N_settlements_500	Number of inhabited human settlements within a 500 m radius area around the apiary	count (1,2, …N)
N_settlements_2000	Number of inhabited human settlements within a 2000 m radius area around the apiary	count (1,2, …N)
**3. Random factors**		
ID	Identification code of each apiary	categorical
Year	Specific year of the study period (2006, 2007 or 2008)	categorical

### Data analysis

We built binomial or negative binomial Generalized Linear Mixed Models (GLMM) to test how different environmental factors, apiary features and spatial and temporal (yearly) patterns affect the probability of damage to an apiary and, in case of damage, the number of beehives attacked (damage intensity). First, we tested for the effects of spatial proximity and intensity of damage in the nearest neighbouring apiary on the probability of an apiary being damaged. For this analysis, we used the data from 2006 to 2008. Second, we considered the variables retained in the previous step, to test whether or not the apiary had been damaged the year before (temporal factor) and the intensity of that damage. For this analysis, we used the data from 2007 and 2008. Third, we added the environmental variables and apiary features to analyse their potential effect on the probability of an apiary being damaged and on the intensity of the damage. Because the latter analyses also included temporal predictors, we used again the data from the last two years of our study (2007–2008).

We considered the following characteristics of the apiary and its environment at different spatial scales: number of beehives and presence and number of prevention measures; distance to the nearest damaged apiary and its damage intensity; whether a given apiary had been damaged or not the previous year and the number of damaged beehives; percentage cover of scrubland, forest and human settlements (including infrastructures) within a 30 m around the apiary; forest cover, number of human settlements and length (in km) of infrastructures within a 500 and 2000 m around the apiary (Table 1). We included apiary identification and year (in the spatial model) as random factors. We tested for collinearity among variables and model selection was based on Akaike Information Criterion corrected for small sample sizes (AICc; [33]). We run all potential models with the potential combinations of explanatory variables in each of the analyses, i.e., the spatial analysis, the spatial and temporal analyses and, finally, the analyses including spatial, temporal and environmental and apiary-related factors. Model averaging was carried out only for those models with ΔAICc <2 to calculate parameter coefficients and the relative importance values (RIV) of each explanatory variable [33]. Parameter estimates from model averaging derives from weighted averages of these values across all of the considered models [34]. The relative importance value (RIV) of each explanatory variable was estimated by summing Akaike weights across all models that contain the variable [33]. All statistical analyses were performed in R 3.0.2 [35] using lme4 [36] and MuMIn [37] packages.

This study did not require ethics permission under European and Spanish legislation. Permission for fieldwork was granted by Resolution of the General Direction of Biodiversity and Landscape (Department of Environment and Rural Development; Ref. Pres. PA 2007: 18.07-443F-610.000). Apiaries were located in communal and public lands, with no specific requirement for visit permissions.

## Results

**Fig 3:**
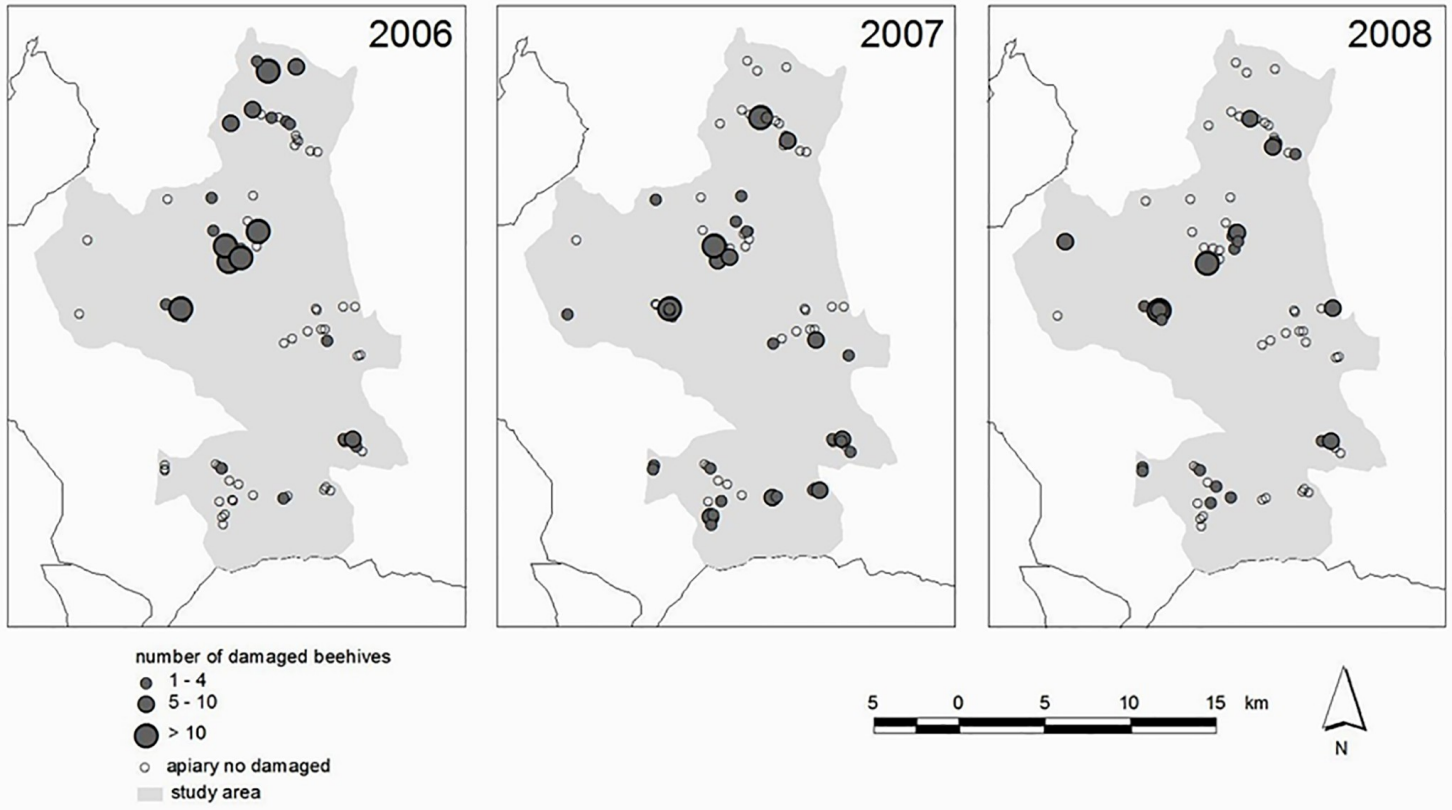
Locations of damaged and undamaged apiaries in the study area in 2006–2008. For damaged apiaries, the number of damaged beehives is also shown.

Between 2006 and 2008, 2,269 beehives were damaged by brown bears in the western Cantabrian Mountains. A total of 489 damaged beehives included in 65 apiaries were reported in our study area (346 km^2^), out of which we were able to identify the exact location of 435 beehives (88.9%) in 61 apiaries (Table 2, Fig 3). Four apiaries were not found during fieldwork in the recorded geographic coordinates, suggesting that those apiaries were removed from the locations where they were damaged before fieldwork was conducted. Full data set in S1 Table.

**Table 2:** Descriptive statistics of the main variables recorded in the study.

Variables	Values for damaged apiaries Mean ± SD (range) (N)	Values for undamaged apiaries Mean ± SD (range) (N)		N total	
Number / Intensity of bear damages (year = 2006)	6.7 ± 7.9 (1–33) (N = 26)	- (N = 55)		81	
Number / Intensity of bear damages (year = 2007)	4.5 ± 4.8 (1–25) (N = 33)	- (N = 48)		81	
Number / Intensity of bear damages (year = 2008)	4.3 ± 3.7 (1–13) (N = 26)	- (N = 55)		81	
**Predictors** **1. Effects of spatial closeness/damage intensity of neighbour apiaries on damaged apiaries**					
Distance nearest (year = 2006; km)	1.0 ± 1.3 (0.0–5.8)	1.6 ± 1.3 (0.0–5.9)		81	
Distance nearest (year = 2007; km)	1.0 ± 1.3 (0.0–5.9)	1.0 ± 1.0 (0.0–4.3)		81	
Distance nearest (year = 2008; km)	1.1 ± 1.2 (0.0–4.1)	1.5 ± 1.6 (0.0–6.7)		81	
Intensity nearest (year = 2006)	7.0 ± 8.1 (1–33)	4.4 ± 4.5 (1–26)		81	
Intensity nearest (year = 2007)	4.9 ± 4.8 (1–25)	5.7 ± 5.2 (1–25)		81	
Intensity nearest (year = 2008)	4.7 ± 3.9 (1–13)	5.0 ± 3.4 (1–13)		81	
**2. Effects of damage intensity the year before on damaged apiaries**					
N_apiaries damaged year before (year = 2007)	13 (N = 33)	13 (N = 48)		81	
N_apiaries damaged year before (year = 2008)	11 (N = 26)	22 (N = 55)		81	
Number / Intensity -1 (year = 2007)	3.58 ± 7.72 (0–33)	1.17 ± 2.55 (0–12)		81	
Number / Intensity-1 (year = 2008)	2.19 ± 3.74 (0–13)	1.67 ± 3.83 (0–25)		81	
**3. Apiary features and landscape attributes surrounding the apiary**				
N_apiaries with prevention measures	33 (N = 44)	15 (N = 20)		64	
N_prevention	1.1 ± 0.8 (0–3)	1.4 ± 0.9 (0–3)		64	
N_beehives	20.3 ± 16.9 (1–70)	18.7 ± 23.8 (1–90)		64	
Forest_30 (%)	29.5 ± 25.1 (0.0–90.0)	34.8 ± 24.3 (0.0–90.0)		64	
Scrub_30 (%)	39.5 ± 30.8 (5.0–100.0)	37.6 ± 23.6 (5.0–90.0)		64	
Human_30 (%)	6.8 ± 15.6 (0.0–50.0)	4.5 ± 11.6 (0.0–65.0)		64	
Forest_500 (ha)	19.6 ± 13.6 (3.9–46.4)	20.8 ± 14.5 (1.4–56.4)		64	
Forest_2000 (ha)	337.0 ± 192.4 (110.0–746.4)	420.0 ± 214.2 (140.7–943.7)		64	
Infrastructures_500 (km)	0.6 ± 0.8 (0.0–3.0)	0.6 ± 0.8 (0.0–3.0)		64	
Infrastructures_2000 (km)	3.1 ± 2.7 (0.0–12.0)	3.1 ± 2.2 (0.0–8.0)		64	
N_settlements_500	1.5 ± 1.0 (0.0–4.2)	1.8 ± 1.0 (0.0–4.5)		64	
N_settlements_2000	15.3 ± 6.4 (7.6–29.8)	17.8 ± 6.0 (7.8–27.4)		64	

The mean number of beehives damaged per apiary and year in these 61 apiaries was 5.1 (range 1–33; Fig 3; see annual intensity of bear damages in Table 2). During 2008, the last year of our study, bears damaged an average of 26.8% (SD = 27.0) of the beehives that were inside the studied apiaries. Forty-three out of the 61 apiaries (70.5%) were damaged only one year, whereas 12 and 6 apiaries were damaged in two and three years, respectively.

**Fig 4:**
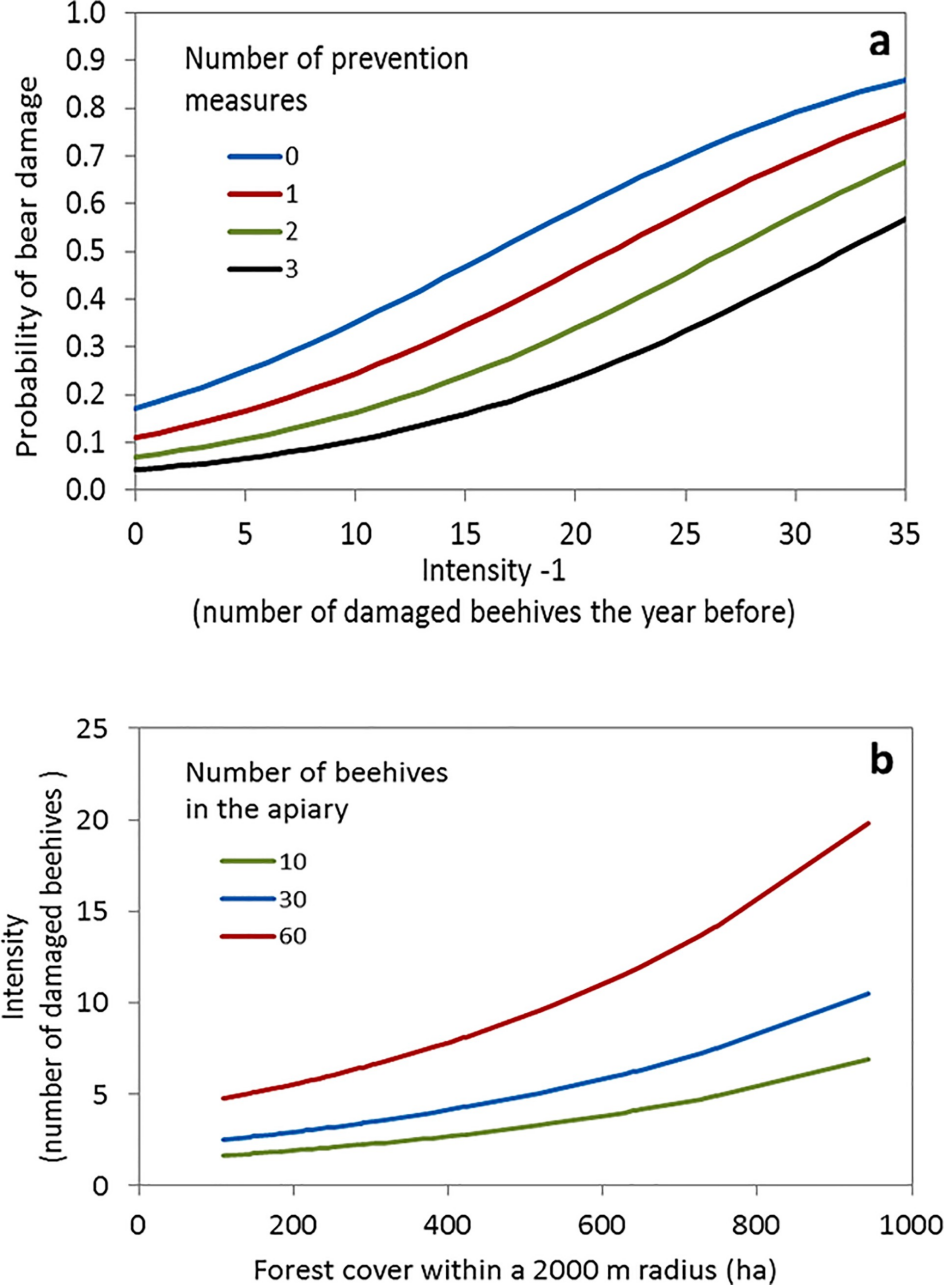
Relationship between (a) the probability of bear damage to an apiary and the number of damaged beehives in the apiary in the previous year and, (b) the intensity of the attack, defined by the number of damaged beehives, and forest cover within a 2000 m radius. Plots are based on the model-averaged parameters (Table 4) that explained the probability and intensity of damage to apiaries by brown bears in the Cantabrian Mountains, Spain. Other variables not represented here were fixed to their mean values and for the year 2007.

Model averaging of the analysis including spatial factors retained the two variables initially considered: the spatial proximity (distance) and the damage intensity on the nearest damaged apiary on the probability of an apiary being damaged (Table 3). Likewise, the analysis adding temporal factors retained the three included variables, i.e., the spatial predictors, the damage intensity of the focal apiary the year before, and the year effect (Table 3). Finally, we added predictors describing environmental factors and apiary features to analyse the overall probability of damage and damage intensity. The most comprehensive model (Table 4) indicated that the probability of an apiary being damaged in a given year was positively related to the intensity of the damage it suffered the year before, i.e., not only if it had been attacked, which suggests a pattern of temporal (annual) autocorrelation in damage occurrence. Against our prediction, the probability of damage was also positively related to the distance to the nearest damaged apiary, i.e., the farther the neighbouring damaged apiary, the higher the probability of an apiary being damaged. The probability of damage was negatively related to the number of prevention measures and the intensity of the damage suffered by the nearest damaged apiary (Fig 4). Both the spatial and the temporal components were significant and showed high RIV values (RIV = 0.83 and 0.78, respectively; Table 4).

**Table 3:** Model-averaged coefficients and relative importance values (RIV) for the variables included in the selected models to analyze the probability of damage on apiaries by brown bears, and the intensity of damage, in the Cantabrian Mountains, Spain. The selected models with ΔAICc<2 that we used for model averaging can be seen in S2 Table. The first model only included spatial variables, and the second model included spatial and temporal variables. In the second model, analyses were restricted to 2007 and 2008 to be able to include variables describing damages in the previous year.

**Effects of spatial proximity and damage intensity of neighbouring apiaries on the probability that an apiary is damaged** *(binomial function; ID and Year as random factor; n = 243)*				
explanatory variables	β	SE	*p*	RIV
(Intercept)	-0.6659	0.1680	0.0001	
Distance nearest	-0.3622	0.1620	0.0253	1.00
Intensity nearest	0.0119	0.0277	0.6670	0.28
**Effects of spatial proximity and damage intensity of neighbouring apiaries, and intensity of damages the year before, on the probability that an apiary is damaged** *(binomial function; ID as random factor; n = 162)*				
explanatory variables	β	SE	*p*	RIV
(Intercept)	-0.5815	0.2636	0.0274	
Intensity-1	0.0735	0.0394	0.0624	0.89
Year (2008)	-0.3704	0.3357	0.2698	0.28
Intensity nearest	-0.0432	0.0416	0.2984	0.28
Distance nearest	0.1784	0.1648	0.2790	0.17

**Table 4:** Model-averaged coefficients and relative importance values (RIV) for the variables included in the selected models to analyze the probability of damage on apiaries by brown bears, and the intensity of damage, in the Cantabrian Mountains, Spain. This model included spatial-, temporal-, environmental-, and apiary-related factors. See S2 Table for further information on the different variables and the selected models with ΔAICc<2 that we used for model averaging). This analysis was restricted to visited apiaries and to 2007 and 2008, to be able to include variables describing damages in the previous year.

**Probability that an apiary is damaged** *(binomial function; ID as random factor; N = 128)*				
explanatory variables	β	SE	*p*	RIV
(Intercept)	-0.392	0.887	0.659	
Intensity-1	0.097	0.048	0.042	1.00
N_prevention	-0.512	0.257	0.046	0.94
N_settlements_500	0.716	0.362	0.048	0.84
Distance nearest	0.443	0.230	0.054	0.83
Intensity nearest	-0.123	0.069	0.076	0.78
Infrastructures_2000	-0.476	0.251	0.059	0.70
Year [2008]	-0.686	0.447	0.125	0.44
Human_30	0.030	0.019	0.127	0.42
Scrub_30	-0.013	0.010	0.203	0.18
Forest_2000	-0.345	0.273	0.206	0.09
Forest_500	0.176	0.238	0.459	0.03
**Intensity of bear damage** *(negative binomial function; ID as random factor; N = 49)*				
explanatory variables	β	SE	*p*	RIV
(Intercept)	1.099	0.339	0.001	
N_beehives	0.212	0.074	0.004	1.00
Forest_2000	0.374	0.153	0.015	0.87
N_settlements_500	-0.391	0.182	0.031	0.77
Intensity-1	0.027	0.017	0.102	0.45
N_prevention	-0.255	0.159	0.108	0.36
Forest_500	0.247	0.104	0.017	0.13

Overall, forty-seven percent of apiaries had stonewalls, 37% had electric fences, and 16% had a wire mesh fence or other prevention measure. The proportion of apiaries without prevention measures, or with one, two and three measures was 23%, 44%, 27% and 6%, respectively (Table 2). The number of prevention measures significantly reduced the probability of the apiary being damaged (RIV = 0.94; Table 4). The number of human settlements within a 500 m radius area around the apiary was positively correlated with the probability of the apiary to be damaged (RIV = 0.84; Table 4). However presence of human infrastructure (i.e., paved and unpaved roads) within a 2,000 m radius area had a negative relation with the probability of damage (RIV = 0.70; Table 4).

Finally, the intensity of bear damages to apiaries was positively related to the number of beehives in the apiary and to forest cover in the 2000 m around the apiary. The intensity of damage was negatively influenced by the presence of human settlements around the apiary (RIV> 0.70 for all of these variables; Table 4, Fig 4).

We were able to visit in the field 44 of the 61 damaged apiaries, plus 20 other apiaries not damaged by bears in 2006–2008, according to the official dataset on bear damages. Environmental attributes, measured at 500 and 2000 m radius around the apiaries, did not differ between visited (n = 44) and not visited apiaries (n = 17) (U Mann-Whitney tests, all P>0.1). Therefore, we assumed that the set of apiaries considered in this study was representative of the apiaries damaged by bears in the area.

## Discussion

Our findings suggest that the patterns of bear depredation on apiaries in the Cantabrian Mountains is modulated by complex relations among different factors, including the yearly recurrence and spatial patterns of attacks, the influence of different characteristics of the apiaries (e.g., the number of preventive measures and the number of beehives per apiary), and the environmental characteristics around the apiaries. Different studies have evaluated how different factors impact on the probability and intensity of depredations by large carnivores (see [38, 39] and references therein), but fewer of them have analysed the role of multiple factors of diverse nature [18], particularly for bears [24].

The probability of an apiary being damaged by brown bears in a given year was positively related to the intensity of damages suffered by the same apiary the year before, yet the probability of damage was lower if the apiary was protected by multiple preventive measures (Fig 4). Nevertheless, the effectiveness of protecting a previously attacked apiary is limited, especially when the attack was intense (Fig 4A). For instance, an unfenced apiary where five beehives were damaged the year before has an attack probability of 0.25 in a given year. However if the attack the previous year damaged 25 beehives, the probability of damage in the focus year increases to 0.69. The installation of a prevention measure decreases this probability to 0.58, and three prevention measures would reduce that probability to 0.33. Our results indicate that it is much more effective to protect the apiaries before an attack occurs, substantially reducing later risks, and reinforce that using several preventive measures is important to prevent damages [40].

In our study area, as in many other places in Europe, there is no obligation to adopt preventive measures, even for claiming compensations for bear damages on apiaries. Then, the tendency of bears to prey on beehives, the difficulty of preventing attacks in some cases, and the positive rewards following a bear intrusion into an apiary could help explain why the probability of an apiary being damaged was positively related to the intensity of damage suffered the year before. Previous results from this study area showed that most of the damages were caused by male bears [41], whose relatively stable home ranges [42] may help explain why some apiaries were repeatedly damaged over time.

The probability of damage was positively related with the number of settlements in a 500 m radius and negatively with the density of roads in a 2000 m radius (Table 4), which likely reflects that beehives are often close to remote and isolated inhabited human settlements, generally inclusive of just a few constructions. The probability of damage increased with distance to the closest damaged beehive and was lower with higher intensity of damage in the closest beehive, suggesting that an attacked beehive prevented damages to the neighbouring ones. Likewise, a recent study [43] found that a reduction in the intensity of wolf *(Canis lupus)* depredations in a given site, in response to lethal management, was related to an increase in the surrounding area. Finally, the observed inter-annual variation in damage probability suggests that yearly variation, e.g., in the availability of natural food resources [44] and/or variation in habitat use by bears, with potential individual variation across bears and years, may have an effect on the probability of bear damage in a given year [41, 45, 46].

Damage intensity was higher for larger apiaries and with more forest cover in the immediate proximity (within 500 and 2000 m radius), whereas it was lower if there were more settlements in the surroundings (Table 4). However, the intensity of the damage was proportionally greater in the smaller apiaries (Fig 4B). For example, apiaries of 60 and 30 beehives surrounded by forest (400 ha in 2000 m radius) would suffer an intensity of attack that would affect, approximately, 8 and 4 beehives respectively (13% of the total number of beehives). However, an apiary of 10 beehives would have 3 beehives damaged, i.e., 30% of the total. This might be due to a satiation effect of the bear visiting the apiary. Therefore, bears use beehives near remote settlements, i.e., the probability of damage was positively affected by number of settlements in the surroundings, but bears made a less intense use of damaged beehives in those areas when these apiaries were surrounded by more settlements. Most intense damage occurred in areas with higher vegetation cover and lower human presence. Human presence influences the distribution and activity patterns of many carnivore species [47, 48, 49, 50], and the relationship between vegetation cover and bear damage occurrence reinforces the importance of protective cover for bears and other large carnivores in human-modified landscapes [51, 52]. Within their home ranges, brown bears use areas that minimize human-caused disturbance, both in terms of movement patterns [29, 53] and resting behaviour, i.e., selecting the most concealed resting sites when bears are close to human settlements [51]. Vegetation cover is indeed an important factor that favours large carnivore persistence, but it also favors damages by carnivores to human property in human-dominated landscapes. This finding applies for several species of canids, felids, and bears in different continents (e.g. [18, 24, 54, 55, 56]).

### Conservation and management implications

We documented that the use of damage prevention measures as traditional stonewalls and/or electric fences (see Fig 2) was associated to low occurrences of damages, especially when different prevention measures were used in combination (Table 4). The application of prevention measures reduced both the probability of bear damage and, to a lower extent, the intensity of the damages (Table 4, Fig 4). Therefore, the implementation of damage preventions measures is a straightforward recommendation to reduce bear damages, especially in the new apiaries, before the bears have preyed on them and thus have a tendency to repeat the attack later, even if preventive measures are used. Furthermore, remote forested sites should be avoided as locations for beehives, to minimize bear damages. We also showed that an attacked apiary prevented damages to the closest ones. Then, according to other studies [43], it is possible that the reduction of attacks in an apiary can increase attacks in a neighbour one.

Application of prevention measures to reduce the impact of carnivores on human property is indeed a well-established, worldwide management recommendation (e.g. [1, 18, 56]). Electrified fences have been suggested as effective tools to reduce bear damages to apiaries [17, 19, 20, 40, 57, 58]. These results and management implications can be extrapolated to the whole Cantabrian area because, (a) our results agree with previous studies carried out in the Cantabrian range [40] and, (b) the relatively large amount of apiaries visited in this study make it representative. In the Department of Rural Development of the Regional Government of Asturias, the official dataset of 2017 registered 90 apiaries in the study area. We located 81 of those apiaries, and 4 that had not even been registered, which illustrates the representativeness of our study.

Increasing available funding to subsidise the cost of installing electric fences around beehives and to maintain them over time would reduce the number of bear depredations on apiaries and the budgetary investment by authorities in damage compensations. Between 2006 and 2008, the Regional Government of Asturias spent ca. €120 000 per year to compensate for damages by bears on apiaries. Protecting beehives in the most risky and new locations, i.e., close to patches of thick vegetation, should be particularly prioritized. Electric fences are relatively easy to maintain and economical to build (a cost of 750 € in raw materials was required to build a photovoltaic energized mesh fence and 450 € for the energized wire fence, 2013–2015 prices [40]), but they require routine inspection and maintenance to ensure proper protection [40, 57, 58]. Similarly, we recommended the use of preventive measures after an attack occurred, e.g., by conditioning future economic compensations to the implementation and proper maintenance of preventive measures.

Human tolerance towards the presence of large carnivores can influence conservation and management goals and decisions, especially in areas with dense human populations [11, 59, 60]. People experiencing damages have more negative attitudes [61], and this would be expected for apiarists experiencing brown bear damage. The attitude of apiarists can shift towards intolerance when damages occur [19] but protecting both human property and large carnivores is possible if we know the factors that trigger large carnivore damages. Brown bears are slowly recovering in the Cantabrian Mountains [27], like other European large carnivore populations [10]. Our management recommendations, build upon previous studies that promotes the protection of human property, such as livestock or apiaries, as an essential tool to prevent wildlife conflicts especially before they occur (e.g. [1, 56, 62, 63]). The recommendations apply to present bear range and, importantly, to neighbouring areas where the bear population is likely to expand in the future and conflicts may arise. Continuous, long-term monitoring of both bear populations and damage occurrence are also necessary to adjust management interventions in the future.

## Supporting information

S1 Table Full data set. (XLSX)

S2 Table Set of top candidate models (ΔAICc <2) with combinations of the variables that could influence the probability and the intensity of damage of apiaries by brown bears in the Cantabrian Mountains, Spain. Variables include environmental factors, apiary features, and spatial and temporal factors. Note that the variables Probability-1, Prevention, Human_30, Infrastructures_500 and N_settlements_2000 were not included in the candidate models after running collinearity analyses.
(DOCX)

## References

[1] Woodroffe R, Thurgood S, Rabinowitz A. People and Wildlife: Conflict or Coexistence? Cambridge: Cambridge University Press, UK; 2005 10.1016/j.jtbi.2005.03.003

[2] Dickman AJ. Complexities of conflict: The importance of considering social factors for effectively resolving human-wildlife conflict. Anim Conserv. 2010;13: 458–466. 10.1111/j.1469-1795.2010.00368.x

[3] Redpath SM, Young J, Evely A, Adams WM, Sutherland WJ, Whitehouse A, et al Understanding and managing conservation conflicts. Trends Ecol Evol. 2013;28: 100–109. 10.1016/j.tree.2012.08.021 23040462 10.1016/j.tree.2012.08.021

[4] Cardillo M, Purvis A, Sechrest W, Gittleman JL, Bielby J, Mace GM. Human population density and extinction risk in the world’s carnivores. PLoS Biol. 2004;2: 909–914. 10.1371/journal.pbio.0020197 15252445 10.1371/journal.pbio.0020197 PMC449851

[5] Ripple WJA, Estes JA, Beschta RL, Wilmers CC, Ritchie EG, Hebblewhite M, et al Status and ecological effects of the world’s largest carnivores. Science (80-). 2014;343: 1241484 10.1126/science.1241484 24408439 10.1126/science.1241484

[6] López-Bao JV, Bruskotter J, Chapron G. Finding space for large carnivores. Nat Ecol Evol. 2017;1: 140 10.1038/s41559-017-0140 28812694 10.1038/s41559-017-0140

[7] Penteriani V, Delgado M del M, Pinchera F, Naves J, Fernández-Gil A, Kojola I, et al Human behaviour can trigger large carnivore attacks in developed countries. Sci Rep. Nature Publishing Group; 2016;6: 20552 10.1038/srep20552 26838467 10.1038/srep20552 PMC4738333

[8] Røskaft E, Bjerke T, Kaltenborn B, Linnell JDC, Andersen R. Patterns of self-reported fear towards large carnivores among the Norwegian public. Evol Hum Behav. 2003;24: 184–198.

[9] Can ÖE, D’Cruze N, Garshelis DL, Beecham J, Macdonald DW. Resolving Human-Bear Conflict: A Global Survey of Countries, Experts, and Key Factors. Conserv Lett. 2014;7: 501–513. 10.1111/conl.12117

[10] Chapron G, Kaczensky P, Linnell JDC, von Arx M, Huber D, Andren H, et al Recovery of large carnivores in Europe’s modern human-dominated landscapes. Science (80-). 2014;346: 1517–1519. 10.1126/science.1257553 25525247 10.1126/science.1257553

[11] Treves A, Karanth KU. Human-carnivore conflict and perspectives on carnivore management worldwide. Conserv Biol. 2003;17: 1491–1499.

[12] Bautista C, Naves J, Revilla E, Fernández N, Albrecht J, Scharf AK, et al Patterns and correlates of claims for brown bear damage on a continental scale. J Appl Ecol. 2016;54: 282–292. 10.1111/1365-2664.12708

[13] Suryawanshi KR, Bhatnagar YV, Redpath S, Mishra C. People, predators and perceptions: Patterns of livestock depredation by snow leopards and wolves. J Appl Ecol. 2013;50: 550–560. 10.1111/1365-2664.12061

[14] Treves A, Wallace RB, Naughton-Treves L, Morales A. Co-Managing Human–Wildlife Conflicts: A Review. Hum Dimens Wildl. 2006;11: 383–396. 10.1080/10871200600984265

[15] Nellemann C, Støen OG, Kindberg J, Swenson JE, Vistnes I, Ericsson G, et al Terrain use by an expanding brown bear population in relation to age, recreational resorts and human settlements. Biol Conserv. 2007;138: 157–165. 10.1016/j.biocon.2007.04.011

[16] Dorresteijn I, Hanspach J, Kecskés A, Latková H, Mezey Z, Sugár S, et al Human-carnivore coexistence in a traditional rural landscape. Landsc Ecol. 2014;29: 1145–1155. 10.1007/s10980-014-0048-5

[17] Breck SW, Lance N, Callahan P. A Shocking Device for Protection of Concentrated Food Sources from Black Bears. Wildl Soc Bull. 2006;34: 23–26.

[18] Miller JR. Mapping attack hotspots to mitigate human–carnivore conflict: approaches and applications of spatial predation risk modeling. Biodiversity and Conservation. 2015; 24(12): 2887–2911.

[19] McKinley BK, Belant JL, Etter DR. American black bear–apiary conflicts in Michigan. Human-Wildlife Interact. 2014;8: 228–234.

[20] Otto TE, Roloff GJ. Black bear exclusion fences to protect mobile apiaries. Human-Wildlife Interactions 2015; 9(1): 78.

[21] Jorgensen CJ, Conley RH, Hamilton RJ, Sanders OT. Management of black bear depredation problems. Workshop on Eastern Black Bear Research and Management 4 1978 pp. 297–321.

[22] Mattson DJ. Human impacts on bear habitat use. International Conference on Bear Research and Management 8 1990 pp. 33–56.

[23] Clark JD, Dobey S, Masters D V., Scheick BK, Pelton MR, Sunquist ME. American black bears and bee yard depredation at Okefenokee Swamp, Georgia. Ursus. 2005;16: 234–244. 10.2192/1537-6176(2005)016[0234:ABBABY]2.0.CO;2

[24] Wilson SM, Madel MJ, Mattson DJ, Graham JM, Burchfield JA, Belsky JM. Natural landscape features, human-related attractants, and conflict hotspots: a spatial analysis of human-grizzly bear conflicts. Ursus. 2005;16: 117–129.

[25] Palomero G, Ballesteros F, Nores C, Blanco JC, Herrero J, García-Serrano A. Trends in Number and Distribution of Brown Bear Females with Cubs-of-the-year in the Cantabrian Mountains, Spain. Ursus. 2007;18: 145–157. 10.2192/1537-6176(2007)18[145:TINADO]2.0.CO;2

[26] Pérez T, Naves J, Vázquez JF, Fernández-Gil A, Seijas J, Albornoz J, et al Estimating the population size of the endangered Cantabrian brown bear through genetic sampling. Wildlife Biol. 2014;20: 300–309. 10.2981/wlb.00069

[27] Martínez Cano I, González Taboada F, Naves J, Fernández-Gil A, Wiegand T. Decline and recovery of a large carnivore: environmental change and long- term trends in an endangered brown bear population. Proc R Soc B. 2016; 9. 10.1098/rspb.2016.1832 27903871 10.1098/rspb.2016.1832 PMC5136588

[28] Fernández-Gil A, Naves J, Ordiz A, Quevedo M, Revilla E, Delibes M. Conflict misleads large carnivore management and conservation: Brown bears and wolves in Spain. PLoS One. 2016;11 10.1371/journal.pone.0151541 26974962 10.1371/journal.pone.0151541 PMC4790950

[29] Martin J, Basille M, Van Moorter B, Kindberg J, Allaine D, Swenson JE. Coping with human disturbance: spatial and temporal tactics of the brown bear (Ursus arctos). Can J Zool Can Zool. 2010;88: 875–883. 10.1139/Z10-053

[30] Naves J, Wiegand T, Revilla E, Delibes M. Endangered species constrained by natural and human factors: the case of brown bears in northern Spain. Conserv Biol. 2003;17: 1276–1289.

[31] Wiegand T, Naves J, Stephan T, Fernandez A. Assessing the Risk of Extinction for the Brown Bear (Ursus arctos) in the Cordillera Cantabrica, Spain. Ecol Monogr. 1998;68: 539–570.

[32] Gonzalez EG, Blanco JC, Ballesteros F, Alcaraz L, Palomero G, Doadrio I. Genetic and demographic recovery of an isolated population of brown bear Ursus arctos L., 1758. PeerJ. 2016;4: e1928 10.7717/peerj.1928 27168963 10.7717/peerj.1928 PMC4860320

[33] Burnham KP, Anderson DR. Model selection and multimodel inference: a practical information-theoretic approach. Berlin: Springer; 2002.

[34] Symonds MR, Moussalli A. A brief guide to model selection, multimodel inference and model averaging in behavioural ecology using Akaike’s information criterion. Behavioral Ecology and Sociobiology. 2011; 65(1): 13–21.

[35] R Development Core Team. R: A Language and Environment for Statistical Computing. R Foundation for Statistical Computing, Vienna https://www.r-project.org. [Internet]. Vienna: R Foundation for Statistical Computing; 2016 p. https://www.R-project.org. Available: https://www.r-project.org.

[36] Bates D, Maechler M, Bolker B, Walker S. Fitting Linear Mixed-Effects Models Using lme4. J Stat Softw. 2015;67: 1–48.

[37] Barton K. Package ‘MuMIn”. https://cran.r-project.org/. 2017.

[38] Treves A, Krofel M, McManus J. Predator control should not be a shot in the dark. Frontiers in Ecology and the Environment. 2016; 14(7): 380–388.

[39] Eklund A, López-Bao JV, Tourani M, Chapron G, Frank J. Limited evidence on the effectiveness of interventions to reduce livestock predation by large carnivores. Scientific reports, 2017 5 18;7(1):2097; 10.1038/s41598-017-02323-w 28522834 10.1038/s41598-017-02323-w PMC5437004

[40] Seijas JM, Osorio MA, García F, Muñoz J, Gonzalez LM, Naves J. Effectiveness of Brown Bear damages protection. Measures to protect apiaries in the Cantabrian Mountains. Carniv Damage Prev News. 2016;12: 26–30.

[41] Naves J, Fernández-Gil A, Ordiz A, Pérez Méndez T, Vázquez JF, Albornoz J, et al. Análisis de los daños atribuidos al oso pardo sobre la agricultura y la ganadería en Asturias. Technical Report, Consejería Medio Ambiente, Principado de Asturias. Oviedo, Spain; 2010.

[42] Dahle B, Swenson JE. Home ranges in adult Scandinavian brown bears (Ursus arctos): effect of mass, sex, reproductive category, population density and habitat type. J Zool. 2003;260: 329–335. 10.1017/S0952836903003753

[43] Santiago-Avila FJ, Cornman AM, Treves A. Killing wolves to prevent predation on livestock may protect one farm but harm neighbors. PLoS ONE. 2018; 13(1): e0189729 10.1371/journal.pone.0189729 29320512 PMC5761834

[44] Naves J, Fernández-Gil A, Rodríguez C, Delibes M. Brown Bear Food Habits At the Border of Its Range: a Long-Term Study. J Mammal. 2006; 87: 899–908. 10.1644/05-MAMM-A-318R2.1

[45] Molinari P, Krofel M, Bragalanti N, Majić A, Černe R, Angeli F, et al Comparison of the occurrence of human-bear conflicts between northern Dinaric Mountains and south-eastern Alps. Carnivore Damage Prevention News. 2016; 12: 9–17

[46] Seijas JM, Naves J. Trabajos para la minimización de daños ocasionados por oso pardo (Ursus arctos) a explotaciones apícolas en la Cordillera Cantábrica. Technical Report, Ministerio de Agricultura, Alimentación y Medio Ambiente-TRAGSTEC. Madrid, Spain. 2017.

[47] Kuijper DPJ, Bubnicki JW, Churski M, Mols B, Van Hooft P. Context dependence of risk effects: Wolves and tree logs create patches of fear in an old-growth forest. Behav Ecol. 2015;26: 1558–1568. 10.1093/beheco/arv107

[48] Kolowski JM, Katan D, Theis KR, Holekamp KE. Daily Patterns of Activity in the Spotted Hyena. J Mammal. 2007;88: 1017–1028. 10.1644/06-MAMM-A-143R.1

[49] Beckmann JP, Berger J. Rapid ecological and behavioural changes in carnivores: the responses of black bears (*Ursus americanus*) to altered food. J Zool. 2003;261: 207–212. 10.1017/S0952836903004126

[50] Valeix M, Hemson G, Loveridge AJ, Mills G, Macdonald DW. Behavioural adjustments of a large carnivore to access secondary prey in a human-dominated landscape. J Appl Ecol. 2012;49: 73–81. 10.1111/j.1365-2664.2011.02099.x

[51] Ordiz A, Støen OG, Delibes M, Swenson JE. Predators or prey? Spatio-temporal discrimination of human-derived risk by brown bears. Oecologia. 2011;166: 59–67. 10.1007/s00442-011-1920-5 21298447 10.1007/s00442-011-1920-5

[52] Llaneza L, García EJ, Palacios V, Sazatornil V, López-Bao J V. Resting in risky environments: the importance of cover for wolves to cope with exposure risk in human-dominated landscapes. Biodivers Conserv. 2016;25: 1515–1528.

[53] Ordiz A, Kindberg J, Sæbø S, Swenson JE, Støen OG. Brown bear circadian behavior reveals human environmental encroachment. Biol Conserv. 2014;173: 1–9. 10.1016/j.biocon.2014.03.006

[54] Stahl P, Vandel JM, Ruette S, Coat L, Coat Y, Balestra L. Factors affecting lynx predation on sheep in the French Jura. Journal of Applied Ecology. 2002; 39(2): 204–216.

[55] Athreya V, Srivathsa A, Puri M, Karanth KK, Kumar NS, Karanth KU. Spotted in the news: using media reports to examine leopard distribution, depredation, and management practices outside protected areas in Southern India. PLoS One. 2015 11 10;10(11):e0142647 10.1371/journal.pone.0142647 26556229 10.1371/journal.pone.0142647 PMC4640542

[56] Van Eeden LM, Crowther MS, Dickman CR, Macdonald DW, Ripple WJ, Ritchie EG, et al Managing conflict between large carnivores and livestock. Conservation Biology. 2018; 32(1): 26–34. 10.1111/cobi.12959 28556528 10.1111/cobi.12959

[57] Di Vittorio M, Costrini P, Rocco M, Bragalanti N, Borsetta M. Assessing the efficacy of electric fences to prevent Bear Damage in Italy. Carnivore Damage Prevention News. 2016; 12: 31–37.

[58] Mettler D. How to prevent damages from bears on beehives the practice of the Swiss system. Carnivore Damage Prevention News. 2016; 12: 18–21

[59] Treves A, Wallace RB, Naughton-treves L, Morales A. Co-Managing Human–Wildlife Conflicts: A Review. Hum Dimens Wildl. 2006;11: 383–396. 10.1080/10871200600984265

[60] Teel TL, Manfredo MJ. Understanding the Diversity of Public Interests in Wildlife Conservation. Conserv Biol. 2010;24: 128–139. 10.1111/j.1523-1739.2009.01374.x 19961511 10.1111/j.1523-1739.2009.01374.x

[61] Conover MR. Resolving human-wildlife conflicts: the science of wildlife damage management. Lewis Publishers, Boca Raton, Florida, USA 2002.

[62] Miller JR, Stoner KJ, Cejtin MR, Meyer TK, Middleton AD, Schmitz OJ. Effectiveness of contemporary techniques for reducing livestock depredations by large carnivores. Wildlife Society Bulletin. 2016; 40(4): 806–15.

[63] Ordiz A, Sæbø S, Kindberg J, Swenson JE, Støen OG. Seasonality and human disturbance alter brown bear activity patterns: implications for circumpolar carnivore conservation?. Animal Conservation. 2017; 20(1): 51–60.

