# Supplementary material for: Patterns of brown bear damages on apiaries and management recommendations in the Cantabrian Mountains, Spain
Source: PLoS One. 2018 Nov 28;13(11):e0206733. doi: 10.1371/journal.pone.0206733 (PMC6261554; doi:10.1371/journal.pone.0206733)
Supplement: S2 Table — Variables include environmental factors, apiary features, and spatial and temporal factors. Note that the variables Probability-1, Prevention, Human_30, Infrastructures_500 and N_settlements_2000 were not included in the candidate models after running collinearity analyses. (DOCX) [file pone.0206733.s002.docx]

| *Probability that an apiary is damaged ( binomial function; ID and Year as random factor; n = 243)* | | | | | | | | | | |
| --- | --- | --- | --- | --- | --- | --- | --- | --- | --- | --- |
|  |  | | |  | | |  | | |  |
| Variables code | AICc | | | delta | | | weight | | |  |
| 2 | 316.85 | | | 0.00 | | | 0.72 | | |  |
| 1,2 | 318.74 | | | 1.90 | | | 0.28 | | |  |
|  |  | | |  | | |  | | |  |
| term code: |  | | |  | | |  | | |  |
| 1 | Intensity nearest | | | | | |  | | |  |
| 2 | Distance nearest | | | | | |  | | |  |
|  | | | | | | | | | | |
| *Probability that an apiary is damaged ( binomial function; ID as random factor; n = 162)* | | | | | | | | | | |
|  | |  | | |  | | |  | | |
| Variables code | | AICc | | | delta | | | weight | | |
| 2 | | 214.64 | | | 0.00 | | | 0.27 | | |
| 1,2 | | 215.55 | | | 0.91 | | | 0.17 | | |
| 2,4 | | 215.56 | | | 0.92 | | | 0.17 | | |
| 2,3 | | 215.62 | | | 0.98 | | | 0.17 | | |
| 1,2,3 | | 216.43 | | | 1.79 | | | 0.11 | | |
| (Null) | | 216.54 | | | 1.90 | | | 0.11 | | |
|  | |  | | |  | | |  | | |
| term code: | |  | | |  | | |  | | |
| 1 | | Year | | |  | | |  | | |
| 2 | | Intensity-1 | | | | | |  | | |
| 3 | | Intensity nearest | | | | | |  | | |
| 4 | | Distance nearest | | | | | |  | | |
|  | | | | | | | | | | |
| *Probability that an apiary is damaged (binomial function; ID as random factor; n = 128)* | | | | | | | | | | |
| Variables code | | | AICc | | | delta | | | weight | |
| 2,5,6,7,8,11 | | | 158.41 | | | 0.00 | | | 0.07 | |
| 2,4,5,6,7,8,11 | | | 158.75 | | | 0.35 | | | 0.06 | |
| 1,2,5,6,7,8,11 | | | 158.76 | | | 0.36 | | | 0.06 | |
| 2,5,7,8,11 | | | 159.10 | | | 0.70 | | | 0.05 | |
| 1,2,6,7,8,11 | | | 159.20 | | | 0.80 | | | 0.05 | |
| 2,4,5,6,7 | | | 159.34 | | | 0.93 | | | 0.05 | |
| 1,2,4,6,7 | | | 159.37 | | | 0.96 | | | 0.05 | |
| 1,2,4,5,6,7,8,11 | | | 159.41 | | | 1.01 | | | 0.04 | |
| 1,2,6,7,8 | | | 159.48 | | | 1.07 | | | 0.04 | |
| 2,3,5,6,7,8,11 | | | 159.50 | | | 1.09 | | | 0.04 | |
| 1,2,4,5,6,7 | | | 159.57 | | | 1.16 | | | 0.04 | |
| 2,3,5,7,8,11 | | | 159.68 | | | 1.27 | | | 0.04 | |
| 2,4,5,7,8,11 | | | 159.88 | | | 1.48 | | | 0.04 | |
| 1,2,5,7,8,11 | | | 159.92 | | | 1.51 | | | 0.03 | |
| 2,4,5,6,7,8 | | | 159.95 | | | 1.55 | | | 0.03 | |
| 2,3,5,8,9,11 | | | 159.99 | | | 1.58 | | | 0.03 | |
| 1,2,3,5,6,7,8,11 | | | 160.00 | | | 1.60 | | | 0.03 | |
| 2,5,6,7,8,10,11 | | | 160.16 | | | 1.75 | | | 0.03 | |
| 2,3,5,7,8,9,11 | | | 160.18 | | | 1.77 | | | 0.03 | |
| 2,4,5,6,7,11 | | | 160.22 | | | 1.81 | | | 0.03 | |
| 1,2,4,5,6,7,8 | | | 160.24 | | | 1.83 | | | 0.03 | |
| 1,2,5,6,7,8 | | | 160.29 | | | 1.89 | | | 0.03 | |
| 1,2,4,6,7,8 | | | 160.29 | | | 1.89 | | | 0.03 | |
| 2,5,6,8,11 | | | 160.35 | | | 1.94 | | | 0.03 | |
| 2,4,5,6,7,8,9,11 | | | 160.40 | | | 1.99 | | | 0.03 | |
|  | | |  | | |  | | |  | |
| term code: | | |  | | |  | | |  | |
| 1 | | | Year | | |  | | |  | |
| 2 | | | Intensity-1 | | | | | |  | |
| 3 | | | Scrub_30 | | | | | |  | |
| 4 | | | Human_30 | | | | | |  | |
| 5 | | | Distance nearest | | | | | |  | |
| 6 | | | Intensity nearest | | | | | |  | |
| 7 | | | N_prevention | | | | | |  | |
| 8 | | | N_settlements_500 | | | | | |  | |
| 9 | | | Forest_2000 | | | | | |  | |
| 10 | | | Forest_500 | | | | | |  | |
| 11 | | | Infrastructures_2000 | | | | | | | |
|  | | |  | | |  | | |  | |
| *Intensity of bear damage (negative binomial function; ID as random factor; n = 49)* | | | | | | | | | | |
| Variables code | | | AICc | | | delta | | | weight | |
| 3,4,6 | | | 229.74 | | | 0.00 | | | 0.25 | |
| 2,3,4,6 | | | 230.22 | | | 0.48 | | | 0.20 | |
| 1,2,3,4,6 | | | 230.64 | | | 0.89 | | | 0.16 | |
| 1,3,4,6 | | | 230.68 | | | 0.93 | | | 0.16 | |
| 1,5,6 | | | 231.02 | | | 1.27 | | | 0.13 | |
| 4,6 | | | 231.55 | | | 1.80 | | | 0.10 | |
|  | | |  | | |  | | |  | |
| term code: | | |  | | |  | | |  | |
| 1 | | | Intensity-1 | | | | | |  | |
| 2 | | | N_prevention | | | | | |  | |
| 3 | | | N_settlements_500 | | | | | |  | |
| 4 | | | Forest_2000 | | | | | |  | |
| 5 | | | Forest_500 | | | | | |  | |
| 6 | | | N_beehives | | | | | |  | |
